# Design and Control of Perylene Supramolecular Polymers through Imide Substitutions

**DOI:** 10.1002/chem.202103443

**Published:** 2021-11-05

**Authors:** Robert S. Wilson‐Kovacs, Xue Fang, Maximilian J. L. Hagemann, Henry E. Symons, Charl F. J. Faul

**Affiliations:** ^1^ School of Chemistry University of Bristol Cantock's Close Bristol BS8 1TS UK; ^2^ School of Chemistry University of Bristol Cantock's Close Bristol BS8 1TS UK

**Keywords:** controllable self-assembly, functional materials, molecular design, perylene diimide, supramolecular polymers

## Abstract

The number and type of new supramolecular polymer (SMP) systems have increased rapidly in recent years. Some of the key challenges faced for these novel systems include gaining full control over the mode of self‐assembly, the creation of novel architectures and exploring functionality. Here, we provide a critical overview of approaches related to perylene‐based SMPs and discuss progress to exert control over these potentially important SMPs through chemical modification of the imide substituents. Imide substitutions affect self‐assembly behaviour orthogonally to the intrinsic optoelectronic properties of the perylene core, making for a valuable approach to tune SMP properties. Several recent approaches are therefore highlighted, with a focus on controlling 1) morphology, 2) H‐ or J‐ aggregation, and 3) mechanism of growth and degree of aggregation using thermodynamic and kinetic control. Areas of potential future exploration and application of these functional SMPs are also explored.

## Graphical abstract

**This review covers** design principles to control the structure of perylene diimide (PDI) supramolecular polymers (SMPs) by modifying their imide substituents. How specific designs have been adapted and expanded is discussed, and various strategies to create future PDI motifs for highly controlled, tailored SMPs are highlighted. A focus is put on thermodynamic and kinetic control over 1) morphology, 2) H‐ or J‐aggregation, and 3) mechanisms of growth.

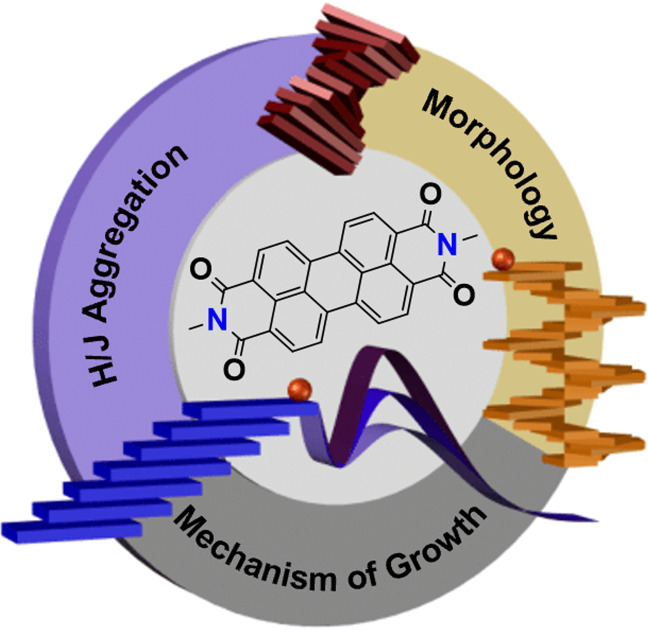

## Introduction

As conjugated, biologically inactive chromophores with excellent thermal, chemical and photostability,^[1]^ perylene diimides (PDIs) ‐ also termed perylene bisimides (PBIs) ‐ have promising applications in a wide range of fields: in solar cells for organic photovoltaics (OPVs),^[2]^ as organic p‐ and n‐type semiconductors,^[3]^ in bulk heterojunction and single‐molecule^[4]^ organic field‐effect transistors (OFETs),^[5]^ logic gates,^[6]^ as light harvesters for artificial photosynthesis^[7]^ and as biological sensors.^[8]^

The key functional groups of PDIs are their conjugated aromatic cores, whose HOMO‐LUMO gaps correspond to wavelengths of 500–700 nm, making these compounds strongly absorbent in the visible region of the electromagnetic spectrum. This region also corresponds to the strongest irradiation of sunlight at sea level, which makes PDIs desirable candidates for OPVs and light‐harvesting applications.^[9]^ Furthermore, the rigidity of the PDI core minimises nonradiative energy loss (e. g., via rotational motion) and its low‐energy triplet excited state minimises intersystem crossing and promotes singlet fission,^[10]^ making PDIs intense fluorophores (up to 100 % quantum yield) with high exciton diffusion lengths (up to 2.5 μm),^[11]^ thus improving their efficiency for photovoltaic and light‐harvesting applications.^[12]^ Flanked by four electron‐withdrawing imide carbonyls, the conjugated cores of PDIs are electron poor, giving them excellent oxidative stability in both their neutral and reduced forms.^[13]^ This stability makes PDIs excellent n‐type charge carriers.^[14]^ Furthermore, PDI cores can also be self‐doped through *bay* and *ortho* substitution with electron‐donating or electron‐withdrawing moieties,^[15]^ allowing their semiconductive behaviour to be tuned, even to exhibiting p‐type semiconductive behaviour.^[16]^ Generally, the rigid PDI core minimises distortion during polaron migration, enabling efficient electron transfer and charge carrier mobilities comparable to those of amorphous silicon.^[17]^ Very importantly, neither the HOMO nor LUMO of PDIs are delocalised to their imide substituents, so imide substitutions of PDIs have minimal effect on the intrinsic optoelectronic properties of disaggregated, unimeric PDI molecules.

The aggregation of PDIs into π‐stacked aggregates heavily influences their resultant optoelectronic properties;^[18]^ it is in this regard that imide substitution can significantly change the optoelectronic properties of PDIs. The offset π‐stacking, mediated via a balance of coulombic interactions between electron‐poor cores and electron‐rich imide carbonyls, causes orbital overlap in PDI aggregates, leading to orbital splitting^[19]^ which modulates the HOMO‐LUMO gap. The HOMO‐LUMO gap can be changed by controlling the geometry and extent of orbital overlap in such π‐stacked structures, for example, through adding sterically‐demanding substituents^[20]^ or by introducing further directed interactions.^[21]^ Changes in the geometry of π‐orbital overlap in a stack also determine whether H‐ or J‐aggregates form,^[22]^ which will be further discussed in the section entitled *“Controlling H‐ or J‐ aggregation”*. Through controlling the aggregation of PDIs, emergent properties can be encoded for through the rational design of PDI motifs.^[23]^

Whilst these properties apply to PDI aggregates in both bulk and low‐dimensional SMP materials, it is the latter which have garnered much interest in the past few years, both within the field of PDIs and the wider field of π‐conjugated SMPs.^[24]^ Their low dimensionality makes them an attractive route for device miniaturisation, particularly in nanoelectronics, nanophotonics, and sensors, opening avenues to create components at the molecular and supramolecular scale with high surfaces areas.^[25]^ Furthermore, SMPs with controlled dimensions allow for the probing of physical properties, including optoelectronic phenomena such as exciton diffusion, waveguiding and charge transport,^[26]^ as well as chemical properties such as solvent‐solute interactions that affect the potential energy surfaces of dynamic systems.^[27]^ Supramolecular polymerisation, in tandem with classical synthetic techniques, can even be used to realise materials with novel physical properties ‐ for example, in the case of covalently ′locked’ (i. e., crosslinked) PDI SMPs that exhibit more stable conduction bands which cannot be accessed in the bulk phase of the unlocked PDIs,^[28]^ and whose optoelectronic properties can be modulated via changing the rigidity of the covalent locker moiety used.

With a range of properties able to be encoded for on the molecular level, PDIs have great potential to be used to create finely tuned optoelectronic materials and devices.^[29]^ However, despite the growing range of methods available to encode for emergent aggregate properties, the complex interplays and trade‐offs that arise from designing supramolecular properties at the molecular level present ongoing challenges. PDIs have a significant advantage over other chromophores owing to their ease of substitution at the imide position ‐ these imide substituents do not modify the HOMO‐LUMO gap of disaggregated molecules and thus can be used to modify the properties of PDI aggregates in a targeted fashion.

**Scheme 1:**
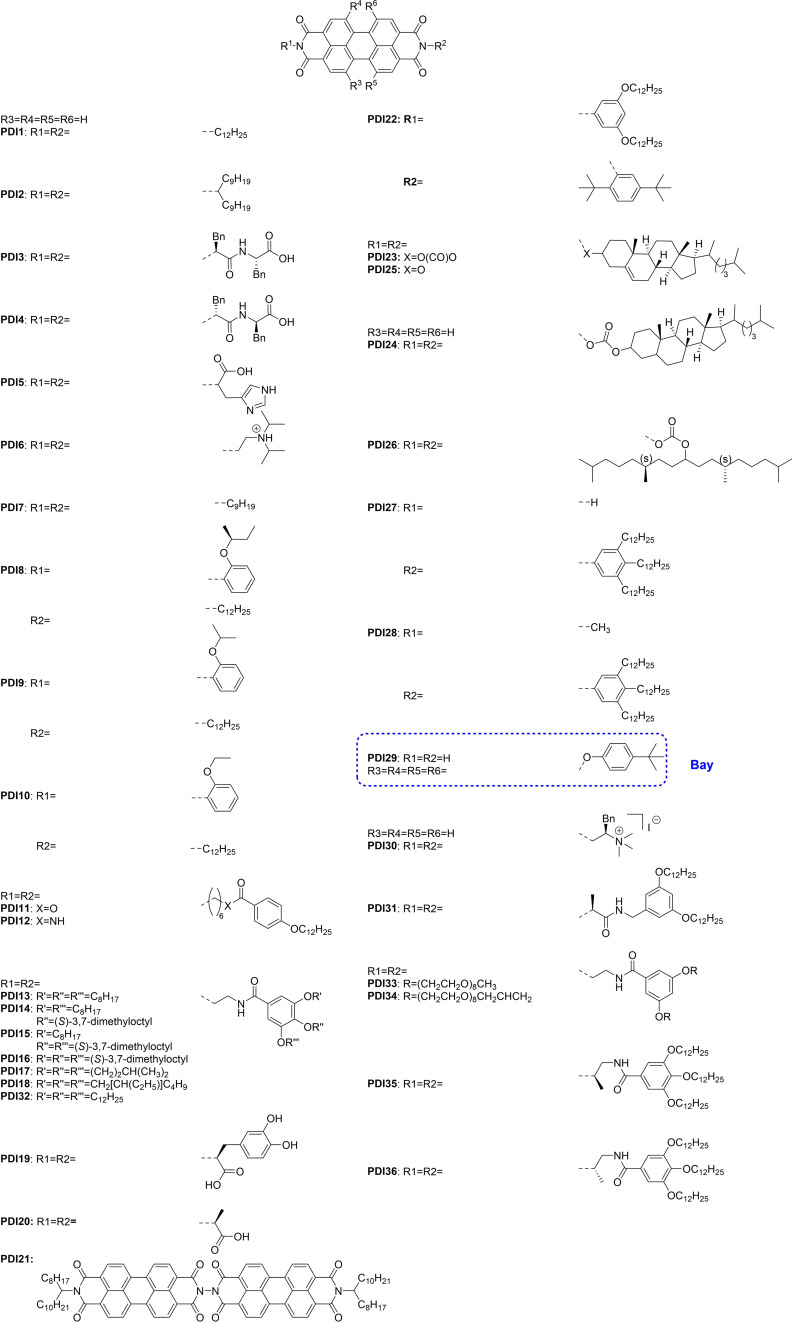
Structures of all PDI compounds described in this review. All compounds are purely imide substituted except for **PDI29**. The imide substituents are identified as “R1” and “R2” and the *bay* substitutions “R3”, “R4”, “R5” and “R6”. Additional substituent groups are marked with “R” or “X”.

As substitution of the perylene core affects both the inherent molecular optoelectronic properties of disaggregated PDIs and the characteristics of their aggregates, discussions covering *core* substitutions are just introduced for one example (**PDI29**). This review therefore covers design principles to control the structure of PDI SMPs by modifying their imide substituents, discusses how specific designs (Scheme 1) have been adapted and expanded, and highlights the viability of various strategies to create future PDI motifs for highly controlled, tailored SMPs. The discussion focusses on three aspects of control in these systems: controlling morphology, controlling H‐ or J‐aggregation, and controlling mechanism of growth.

## Controlling Morphology

For supramolecular systems, it is already well established that careful molecular design can tune the morphology of aggregates and SMPs. A related expression of simple structural hierarchy is most familiar in the packing parameter of colloidal systems, whereby the critical chain length, volume and headgroup area of amphiphiles are used to accurately predict the morphology of the formed self‐assembled structures.^[30]^ Whilst there is no similar comprehensive and generalised relationship established for SMPs, or for that matter PDI‐based SMPs, a wealth of research exists focussed on tuning PDI unimers to control the morphology of the resulting polymers.^[31]^ More recently, PDI SMPs have been developed that also exhibit switchable morphologies.[^31c^, ^32^]

To control the morphology, interactions with solvent and steric properties should be carefully balanced to control the π‐stacking of the perylene cores and interactions of the imide substituents.^[31a]^ The length, volume and presence of additional functional groups all influence the solvophilic properties of side chains and can introduce new intermolecular interactions. Opportunities therefore exist for careful control over the morphology of PDI aggregates, and some of the most recent examples are discussed below.

### The effect of steric hindrance

Whilst predicting PDI aggregation is complicated by the constant presence of significant π‐interactions, the same steric and packing effects that control the morphology of amphiphiles still apply. The steric effect was clearly seen in the morphology of two symmetric alkyl‐disubstituted PDIs prepared by Zang et al.: linear dodecyl (**DD**)‐substituted PDI (**PDI1**) and swallow‐tailed nonyldecyl (**ND**)‐substituted PDI (**PDI2**).^[31a]^ The lengths of both side chains are similar, but the branched configuration of the **ND** moiety enhanced its volume. Due to the steric effect of the branched **ND** side chain, the stacking of the perylene core was distorted and weakened. In a 35/65 (v/v) water/methanol dual‐solvent system, weak π‐stacking of **PDI2** was confirmed by UV/Vis spectroscopy. This weak π‐interaction did not facilitate 1D self‐assembly; instead, **PDI2** formed nanospheres. In contrast to **PDI2**, **PDI1**, with less sterically‐demanding side chains and more favourable π‐stacking, formed 1D nanobelts.^[31a]^

**Figure 1:**
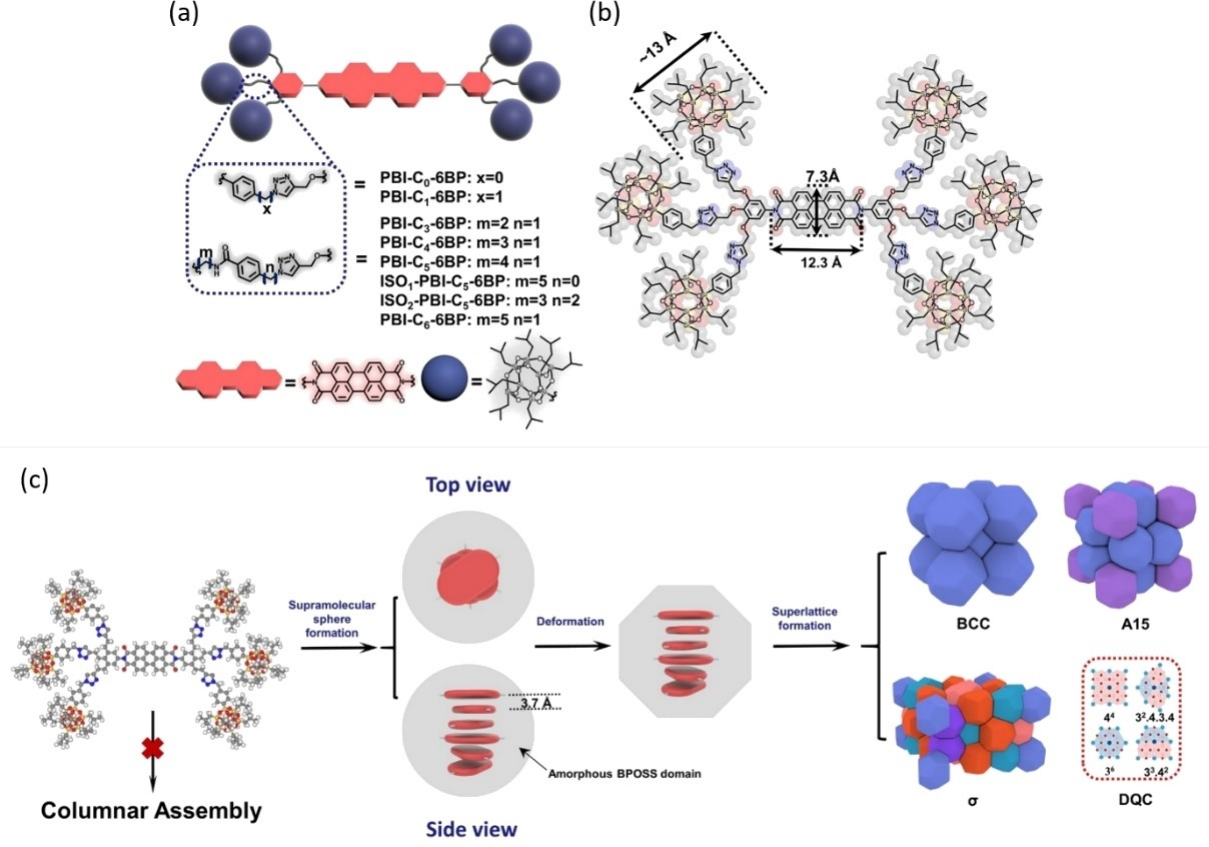
(a) Schematics of BPOSS‐PDIs with various linker molecules. (b) Dimensions of BPOSS cage and perylene core. (c) Schematic showing the formation of SMP superlattices. Adapted with permission from Ref. [34]. Copyright © 2020 Wiley‐VCH GmbH.

In 2020, Huang et al. successfully designed a series of polyhedral oligomeric silsesquioxane (POSS) cage substituted PDIs (Figure 1a), which led to novel spherical packing phases rather than the conventional columnar or lamellar structures. Six cubic POSS cages were functionalized by isobutyl groups, forming rigid peripheric blocks (BPOSS). The BPOSS moieties were connected to the imide positions of the perylene core through a trioxyl phenyl group substituted by linker molecules with various components and lengths. The tendency of BPOSS cages^[33]^ to crystallise was restricted by the flexibility of linkage molecules and π‐stacking interactions of PDI cores, where π‐stacking, as well as the tendency of PDIs to form columnar assemblies, was reversely impeded by the steric hindrance of BPOSS cages (Figure 1b). By tuning the flexibility of linker molecules, diverse SMP superlattices including body‐centered cubic (BCC), A15, σ and decagonal quasicrystal (DQC) phases were formed (Figure 1c). Moreover, an inverse phase transition from BCC to σ phase was achieved by an annealing process.^[34]^

### Solvent‐selective behaviour

**Figure 2:**
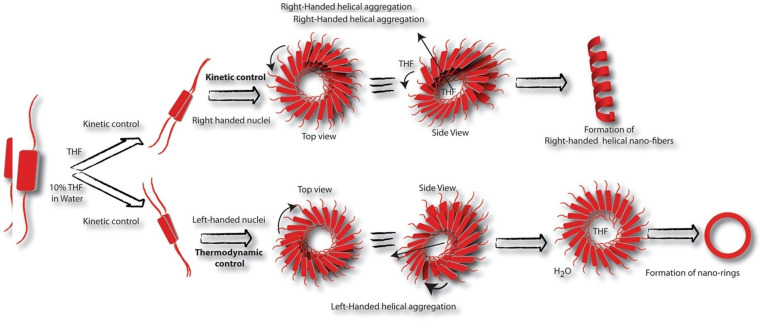
Schematic of thermodynamic and kinetic control based on solvent selective behaviours of diphenylalanine substituted PDIs **(PDI3)** in pure THF and 1 : 9 (v/v) THF/water.^[31c]^ Copyright © 2017, Ahmed et al. (Open access. Springer Nature. Creative Commons CC‐BY).

Solvents play an important role in supramolecular polymerisation:^[27a]^ they can affect π‐stacking and electrostatic attractions, and thus the interplay between these attractive interactions and steric phenomena (as found for the packing parameter). The amphiphilicity of imide substituents, which gives rise to solvent‐selective behaviour, provides a dynamic means to change PDI morphology by modifying solvent conditions. Using a chiral diphenylalanine‐disubstituted PDI (**PDI3**) and its *D*‐analogue (**PDI4**), Ahmed et al. observed reversible morphology changes from nano‐rings to helices, both comprised of J‐aggregating PDIs (Figure 2).^[31c]^

In pure THF, a moderate solvent for the unimers, the assembly process proceeds under kinetic control. In the case of **PDI3**, right‐handed nuclei initially formed, promoting the formation of right‐handed helical nano‐fibres. In contrast, in a THF : water mixture (1 : 9), left‐handed nuclei are formed, which then grow into nano‐rings under thermodynamic control. The same effect was also confirmed in **PDI4** by observing mirror circular dichroism signals in each case. The authors postulate that the formation of stable nano‐rings is due to the expulsion of water (the poor solvent) from the centre of the helix, leaving the good solvent (THF) inside, as indicated by NMR. This partition of solvent creates a critical hydrodynamic radius which acts as a barrier to further growth, preventing the formation of elongated nano‐fibres and preserving the ring‐shaped nuclei.

### Stimuli‐responsive morphology control

**Figure 3:**
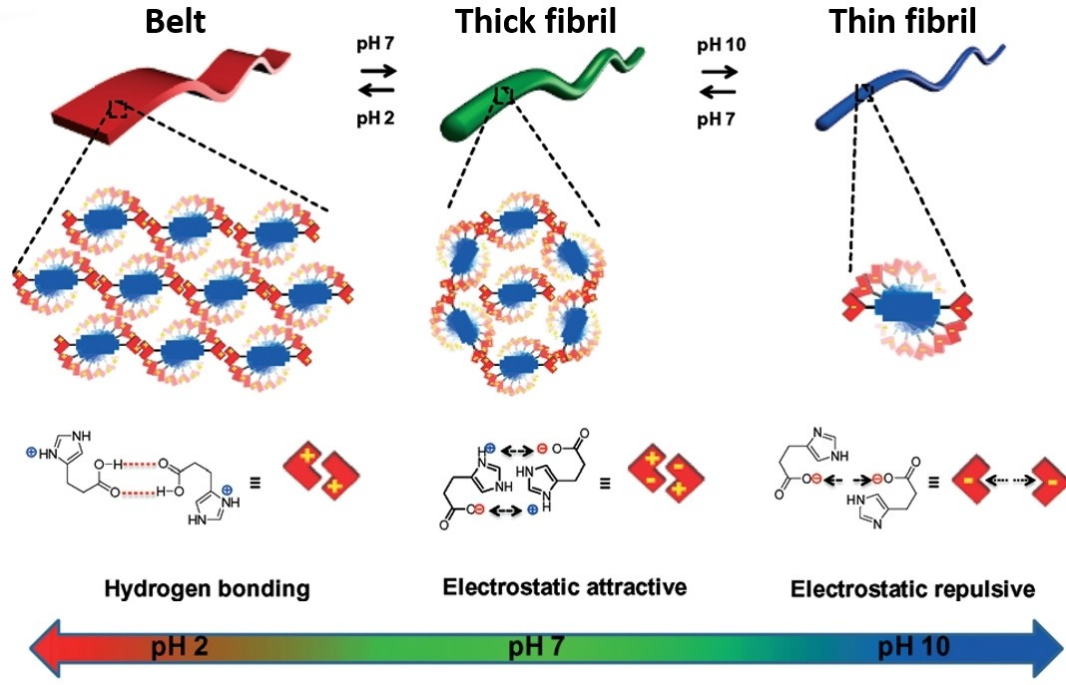
pH‐triggered reversible fibril‐belt switching behaviour via hydrogen bonding (acidic pH), electrostatic attractive interaction (neutral pH), and electrostatic repulsive interaction (basic pH) of histidine‐functionalized PDI (**PDI5**).^[45]^ Reproduced with permission from Ref. [35]. Copyright 2016, Royal Society of Chemistry.

External stimuli can be used to switch the morphology of PDI aggregates, allowing morphology to be controlled in situ. Taking advantage of the weak acidity of an imide‐coupled histidine residue, Pandeeswar and Govindaraju discovered the reversible pH‐triggered fibril‐belt switching behaviour of a histidine‐functionalized PDI (**PDI5**).^[35]^ The supramolecular morphology was reversibly controlled via electrostatic interactions stemming from the histidine carboxylic acid group. At high pH, the carboxylic acid groups within the aggregates were predominantly deprotonated, creating negatively charged fibres that induced greater electrostatic repulsion (Figure 3).^[35]^ Thus, **PDI5** assembled into a uniform thin nanofibril network of micrometre length and a width of 22±1 nm. As the pH decreased, the side groups were gradually protonated, removing these negative charges and minimizing interfibrillar repulsion. As a result, the fibres could grow along their lateral axis, reversibly forming a belt structure.

**Figure 4:**
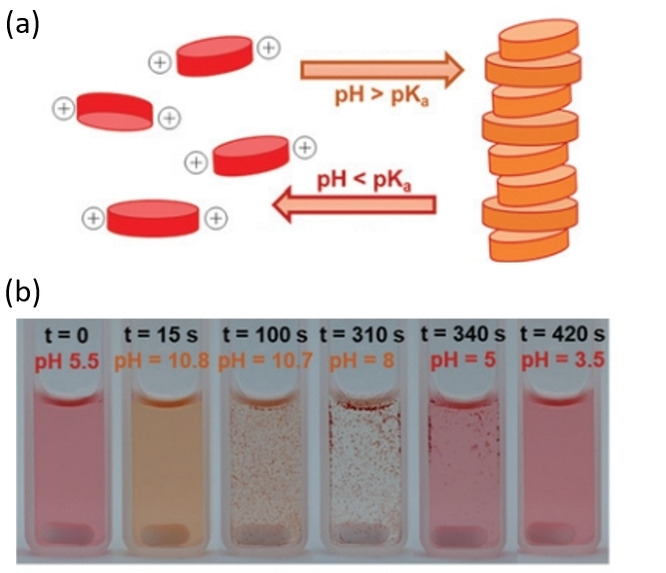
(a) Schematics of pH‐responsive assembly and de‐assembly process of **PDI6**. (b) Reversible colour change on response to circular pH modification. Adapted from Ref. [32]. Royal Society of Chemistry. Creative Commons Attribution 3.0 Unported Licence.

Another pH‐responsive PDI system was presented in a recent study by Panzarasa et al., where a transient assembly based on diisopropylethyleneamine disubstituted PDI (**PDI6**) was controlled by a programmable pH cycle inspired by clock reactions.[^32^, ^36^] The tertiary amine structures were protonated when the pH was less than 6.5, building up electronic repulsion that overcame the π‐stacking (Figure 4a).^[37]^ The aggregation recovered as the pH was increased to basic conditions, highlighting the cyclability of the system. The colour change (Figure 4b) of the controllable transition between discrete unimers and aggregated assemblies could allow potential applications in pH sensors and chemical clock studies.^[32]^

### Multistage aggregates of PDIs

Dynamic control of PDI morphology is also evident in multicomponent aggregates of PDIs. The introduction of other amphiphiles with complementary binding groups, which introduce non‐covalent interactions like hydrogen bonding with the PDI derivatives, disrupts perylene aggregation and favours the self‐assembly of a hierarchical SMP. This behaviour has been examined in detail in the case of PDIs substituted with melamine derivatives, and their complexation with *N‐*dodecylcyanurate (**CA**), a complementary binding motif to melamine.^[38]^ Before the addition of **CA**, these melamine‐PDIs self‐assembled into strongly bound, π‐stacked nanowires, but these aggregates were disrupted when **CA** was introduced to the solution. Hydrogen bonding between the PDI melamine derivatives and **CA** led to incorporation of the cyanurate into the aggregates in a 1 : 1 ratio, creating oligomeric species that no longer exhibited the characteristic PDI π‐stacking absorption shoulder at 540 nm. Increasing the concentration of the **PDI‐CA** solution induced π‐stacking, yielding ribbon‐like aggregates. The authors postulated these aggregates to be bound via perylene π‐stacking along the width of the ribbons, while melamine‐cyanurate hydrogen bonding drove aggregation along the length.

**Figure 5:**
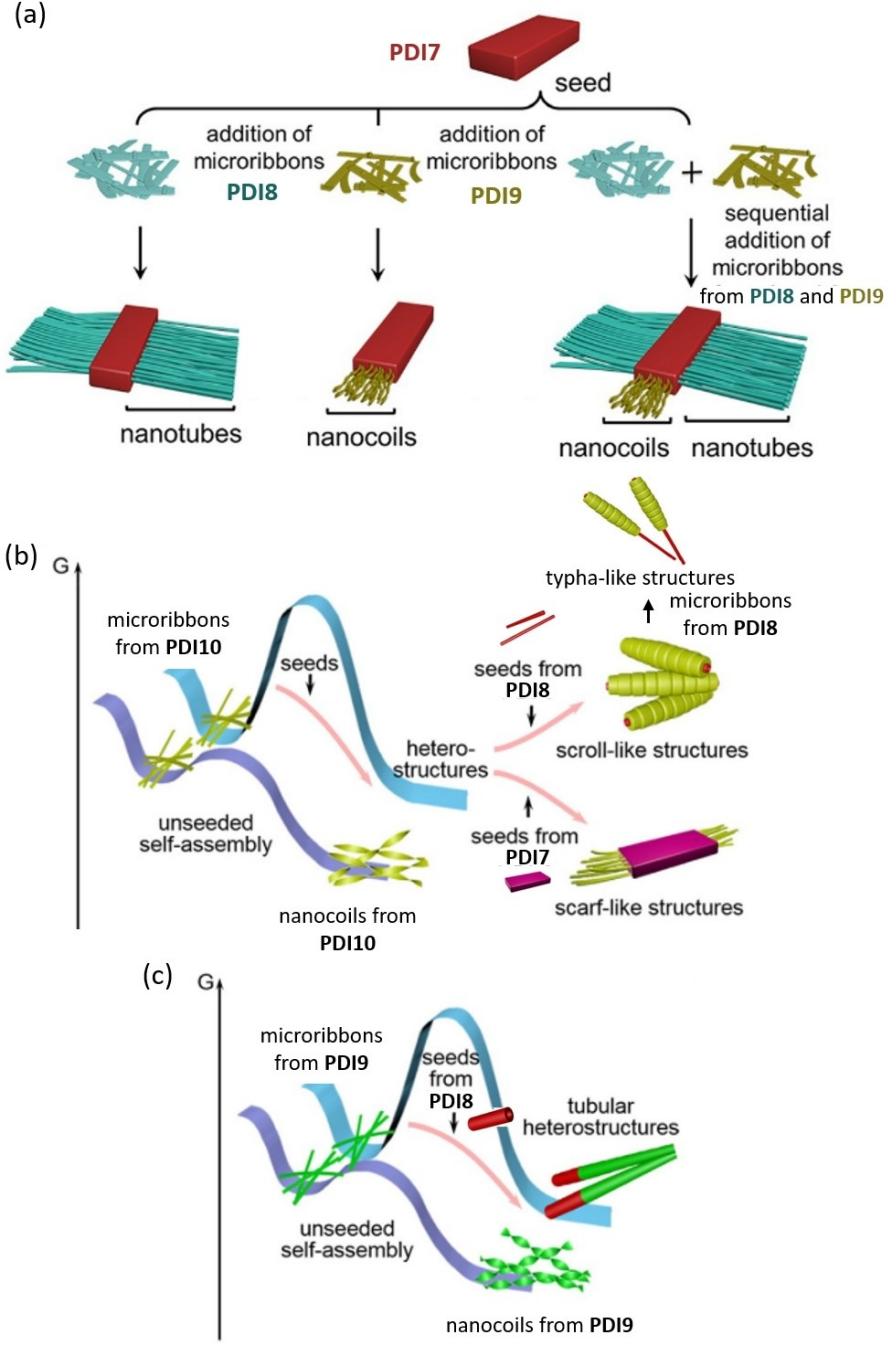
Schematics of (a) the formation of 2D hierarchical PDI heterostructures; pathway comparison between seeded and unseeded polymerisation of (b) **PDI10** and (c) **PDI9**. (a) is reproduced from Ref. [39]. Copyright 2017, Wiley‐VCH. Parts (b) and (c) are reproduced from Ref. [42]. Copyright 2019 Wiley‐VCH.

Liu et al. showed a 2D hierarchical PDI heterostructure obtained via sequential 2D seeded growth of three PDIs (Figure 5a).^[39]^ Details of the seeded growth method are further elaborated on in the *Controlling Mechanism of Growth* section. In Liu's work, a *n*‐nonyl disubstituted PDI (**PDI7**) was first assembled into microribbons seeds by vapour diffusion. A seed solution of **PDI7** was separately added to solutions of two asymmetrically modified PDI unimers (**PDI8, PDI9**) with similar structures. **PDI8** and **PDI9** shared one identical side chain (*n*‐dodecyl chain) but differed in the extent of steric bulk at the other imide position. **PDI8** and **PDI9** were previously revealed to initially form kinetically trapped (metastable) microribbons. After thermodynamic equilibration, the microribbons broke up into nanowires that subsequently formed nanotubes or twisted nanoribbons, which successively formed nanocoils for **PDI8** and **PDI9**, respectively.^[40]^

When **PDI7** was introduced to the system, the microribbon precursor of **PDI8** first nucleated at the edge (long axis) of **PDI7** seeds, growing into parallel rod‐like structures perpendicular to the **PDI7** seed. This growth resulted in 2D heterostructures with a **PDI7** microribbon core and **PDI8** nanotubular branches. In contrast, the microribbon precursor of **PDI9** nucleated at the terminal (short axis) of **PDI7** seeds, followed by helical growth in an epitaxial direction along the seed terminal, resulting in nanocoiled structures. The difference in nucleation site and growth direction of **PDI8** and **PDI9** was explained by lattice matching.^[41]^ Further experiments based on PDI seeds disubstituted with multi‐length alkyl chains revealed that only seeds with long axis intermolecular distances matching the short axis lattice distance of **PDI8** allowed the vertical growth of **PDI8**. Similarities in the lattice structure at the seeds(**PDI7**)**/**unimers(**PDI8**) interfaces of the lattice reduced the nucleation barrier, which thus controlled the nucleation site preference.^[39]^

By sequential addition of the three PDIs, a ternary 2D hierarchical heterostructure was obtained. However, morphological control inhibited due to the mutual interaction between the structurally similar **PDI8** and **PDI9**. Thus, it was proposed that the further design of more complex PDI heterostructures with controllable morphology would ideally be suitable for dissimilar building blocks with less interaction between each other.^[39]^

In 2019, Liu et al. further explored the hetero‐seeding approach to achieve multidimensional hierarchical aggregates.^[42]^ Unprecedented 3D scroll‐like, typha‐like, and scarf‐like nanostructures (Figure 5b) as well as 1D tubular heterostructures (Figure 5c) were achieved by altering seed components and crystalline morphologies. **PDI10** is an analogue of **PDI9** with a less sterically demanding side chain. Both **PDI10** and **PDI9** formed nanocoils via an unseeded pathway. When instead initiated by nanotubular seeds of **PDI8**, the unimers of **PDI10** nucleated at the edge of seeds followed by vertical growth along the side, resulting in 3D scroll‐like **PDI8/PDI10** hetero‐structures. In contrast, **PDI9** unimers grew epitaxially along the seed terminal, forming tubular hetero‐structures. With continued activity and further addition of new unimers to the free interfaces of the seed terminal, the 3D scroll‐like **PDI8/PDI10** heterostructures could further form typha‐like structures when metastable microribbons of **PDI8** were introduced. When **PDI7** microribbons were used as seeds, **PDI10** unimers followed the epitaxial growth along the seed terminal, which eventually led to scarf‐like structures. Interestingly, the typha‐like structures of **PDI8/PDI10**, tubular structures of **PDI8/PDI9** as well as nanocoiled structures of **PDI7/PDI9** were non‐centrosymmetric, further illustrating the significant effects of steric hinderance on morphology control that are mentioned above.

## Controlling H‐ or J‐Aggregation

When organic dyes, including PDIs, undergo π‐stacking, new excitonic bands arise at higher or lower energy levels compared to the bands present in the disaggregated molecules. The position of these bands is determined by the coupling of transition dipole moments between chromophores, which, in turn, is determined by the geometry and overlap of π‐orbitals as they stack. As such these bands are strongly influenced by the arrangement of chromophores in an aggregate.^[43]^ Early theories of molecular exciton coupling of dye molecules, developed by Levinson et al. (1957)^[44]^ and Kasha et al. (1963),^[45]^ generally explained this geometry dependent energy splitting effect. In short, the ‘plane‐to‐plane’ co‐facially arrangement (H‐aggregates) builds up the repulsive interactions between transition dipole moments, which level up the excited state on the basis of the disaggregated state. On the contrary, the ‘end‐to‐end’ offsetting mode (J‐aggregates) decreases the corresponding energy level due to the attraction of the dipole moments. These excitonic bands have profound impacts on the optoelectronic properties of the aggregates, and as such are frequently referred to by their optical shifts relative to the disaggregated molecules ‐ bathochromic (red) shifts for J‐aggregation or hypsochromic (blue) shifts for H‐aggregation.^[46]^

The importance of controlling H‐ or J‐aggregation is related to the favourable optoelectronic properties observed in J‐aggregated PDIs, specifically their enhanced fluorescence quantum yield and excited‐state lifetime compared to H‐aggregates. As such, J‐aggregates are of great interest in optical applications. Though many successful cases of J‐aggregated, *bay*‐substituted PDIs exist,^[47]^ obtaining J‐aggregated PDIs purely through imide‐substitution still presents both challenges and opportunities. Steric hindrance, solvent polarity, pH, concentration and temperature or complexation with additives can all influence whether an SMP undergoes H‐ or J‐aggregation.[^47b^, ^48^]

Through investigating a series of both ester and amide‐substituted PDIs, Jancy and Asha established a correlation between aggregation strength and stacking mode, where both are intimately related to the presence or absence of the hydrogen‐bonding amide moiety.^[49]^ All amide‐substituted PDIs showed strong aggregate peaks in their UV/Vis spectra, while only one ester‐based PDI (**PDI11**) exhibited aggregation at lower temperatures and higher concentrations than the amide series. This result mirrors other findings discussed in this review, where intermolecular amide bonding complements the π‐stacking of perylene cores, increasing binding strength between unimers. In addition, the ester‐substituted **PDI11** formed J‐aggregates with displaced cores, compared to the co‐facial H‐aggregation of amide‐substituted **PDI12**. It was postulated that the inability of ester moieties to undergo intermolecular hydrogen bonding, and thus the absence of a complementary binding force to aid in co‐facial π‐stacking, led to the preferential formation of J‐aggregates.

Whilst H‐aggregation can be promoted by using complementary binding moieties such as the amide group, J‐type aggregation can be achieved through active disruption of co‐facial π‐stacking. In examining the effects of steric substituents on the aggregation behaviour of trialkoxy benzamide‐PDIs (**PDI13‐16**), Ghosh et al. found that PDIs with less sterically demanding (i. e., more linear) chains tend to form H‐aggregates, while PDIs with more sterically demanding (i. e., more branched) chains form J‐aggregates or no aggregates. Co‐facial π‐stacking between perylene cores was found to be weakened when octyl groups were successively replaced with the more sterically demanding (*S*)‐3,7‐dimethyloctyl chains.^[50]^ However, for PDIs exclusively bearing (*S*)‐3,7‐dimethyloctyl chains (**PDI16**), J‐aggregates with similar binding strengths to the octyl‐only substituted PDIs (**PDI13**) were found. This result was due to similar stabilities for H‐ and J‐type stacking for this PDI series, where the successive addition of (*S*)‐3,7‐dimethyloctyl groups (**PDI14** and **PDI15**) led first to disrupted H‐aggregation before full substitution resolved the disrupted stacking and formed J‐aggregates.

**PDI17**, with a shorter isopentyl substituent with disruptive steric bulk but lower entropic loss upon aggregation, confirmed these results. This substituent led to the most stable aggregates studied, and due to co‐facial disruption, formed J‐aggregates. However, if steric disruption was too extreme, aggregation was suppressed completely. In the case of a 2‐ethylhexyl substituted PDI (**PDI18**), only unimeric species could be observed. As a result, fine control over steric hindrance and stacking disruption was required to promote J‐aggregation without suppressing self‐assembly.

In addition to the above‐mentioned two examples, where new molecules were synthesized to achieve different aggregation types, Draper et al. realized control over H‐/J‐aggregation in a single PDI molecule functionalized with *L*‐3,4‐dihydroxyphenylalanine (*L‐*DOPA) at the imide positions (**PDI19**).^[48b]^ The degree of protonation of **PDI19** is tuneable with pH, with the resulting variable electrostatic interactions impacting the aggregates’ self‐assembly behaviour. By comparing the fluorescence intensity and UV/Vis absorbance spectra, it was shown that the fully deprotonated state formed J‐aggregates, whilst the partially deprotonated state resulted in H‐aggregates. The authors also noticed that the type of aggregation modified the second pK_a_ of partially and fully deprotonated **PDI19**, changing from 5.4 to 5.7, respectively. When the pH of both systems was reduced to 3.3, the fully deprotonated **PDI19** (J‐aggregate) formed gels, whilst the partially deprotonated PDI (H‐aggregate) remained in solution. Notably, the mechanism of gelation is not simply due to the aggregation type. Supported by DFT/TD‐DFT calculations and UV/Vis, NMR, rheology and neutron scattering experimental data of an *L*‐alanine disubstituted PDI (**PDI20**) in 2019,^[51]^ Draper's team further concluded that the surface charge on the aggregates was reduced by protonation, the primary factor in decreasing colloidal stability and thus driving gelation.^[51b]^

**Figure 6:**
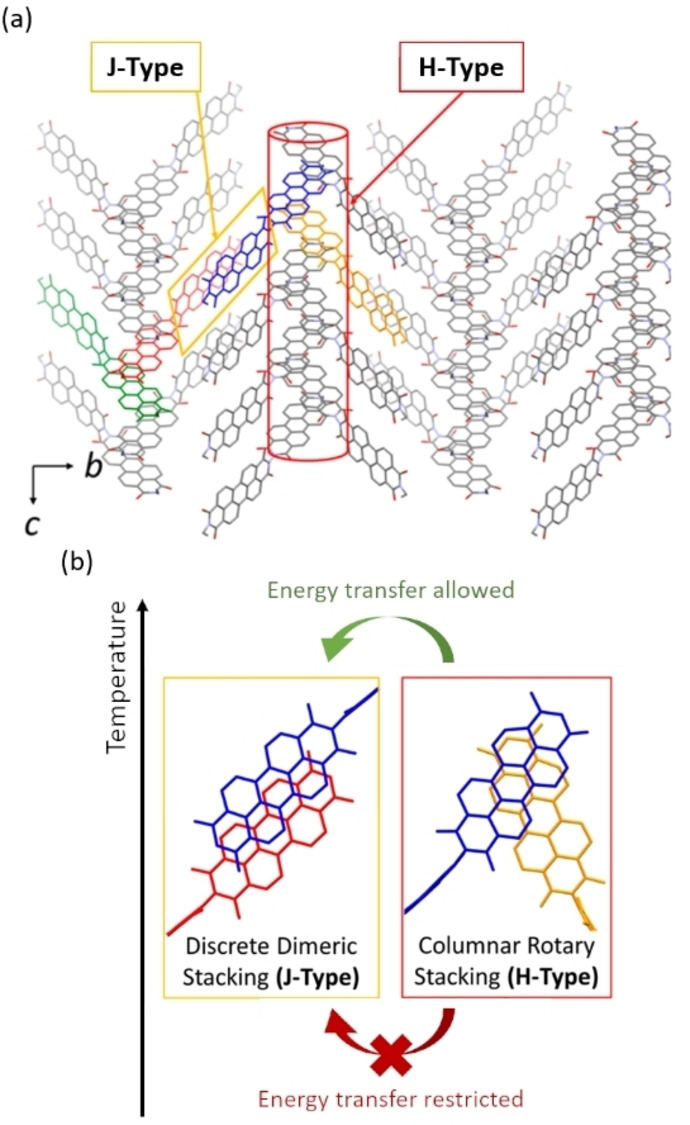
(a) Schematic of H‐/J‐ coexisting aggregation mode in crystalline structure of **PDI21** dyad. (b) Schematics of H‐/J‐aggregated part and temperature‐dependent energy transfer from H‐ to J‐aggregated moieties. Reproduced with permission from Ref. [48]. Copyright 2021, American Chemical Society.

Taking advantage of temperature‐dependent energy transfer from H‐ to J‐aggregates, Chen et al. very recently developed a novel strategy to achieve long‐lived fluorescence with a head‐to‐tail covalently‐linked PDI dyad (**PDI21**).^[48c]^ The imide positions at the termini of each **PDI21** dyad were modified by 2‐octyldodecyl chains. Unprecedently, both columnar rotary stacking (H‐aggregate) and discrete dimeric slipped‐stacking (J‐aggregate) modes coexist in one crystalline structure (Figure 6a). Confirmed by computational analysis, the exciton energy of the H‐aggregated component is higher than the one of the J‐aggregated component. The photoluminescence efficiency at room temperature (300 K) was 12 %, which gradually increased to 90 % at low temperature (80 K). In addition, the fluorescence lifetime increased from 1 to 5 ns (at room temperature) to over 15 ns (below 130 K). This long‐lived fluorescence behaviour was explained by 1) the suppression of non‐radiative energy transfer from H‐ to J‐aggregated moieties (Figure 6b) together with 2) the enhanced vibronically induced transitions at low temperature. This investigation clearly showed the opportunities that can be found by controlling H‐ or J‐aggregation of imide‐substituted PDIs in applications for devices with tunable optoelectronic properties.

As many more complex arrangements and properties gradually emerge in the field of PDI‐based SMPs, continuous efforts have been focussed on more detailed theoretical understanding of the excitonic behaviour in comparison to the Kasha's model.^[52]^ Exclusively focused on long‐range Coulomb coupling, Kasha's model is somehow incomplete due to ignoring the subtle charge transfer and short‐range interactions, which are sensitive to even sub‐angstrom geometrical shift and comparable to the Coulomb coupling in magnitude.[^47a^, ^53^] Such short‐range interactions can interfere with the Coulomb interaction in PDI aggregates,^[54]^ and independently determine the formation of H‐ or J‐aggregates.^[53d]^ The combined effect of long‐range Coulomb coupling and short‐range charge transfer, either constructive or destructive, broadens the general classification of H‐/J‐aggregates into HH‐, HJ‐, JH‐and JJ‐aggregates, where the first letter stands for the contribution from Coulomb coupling, and the second letter represents charge transfer, respectively. The resulting hybrid aggregates display spectroscopic features of both conventional H‐ and J‐ aggregates.^[55]^ When the two contributions are exactly cancelled, the resulting aggregate is called a “null aggregate”, with unimeric spectroscopic features.^[22]^ The null aggregate of PDI was first experimentally proved by Würthner's team in 2018 with a set of *bay*‐substituted PDIs.^[56]^ In 2019, Oleson et al. also showed the control over long‐range dominant (Hj‐) and short‐range dominant (hJ‐) aggregates of PDIs with phenyl‐disubstituted (*imide*) or tetrasubstituted (*bay*) PDIs. Interestingly, the photoluminescence of hJ‐aggregates formed by the *bay*‐substituted PDI is independent of temperature, which is different from the Hj‐aggregates formed by the imide‐substituted version.^[47a]^ Although to date there is still a lack of cases with purely imide‐substituted PDIs exhibiting hybrid H‐/J‐aggregation control, this strategy (i. e., tuning exciton bandwidth based on the contributions from both long‐range and short‐range coupling), provides new insight into designing imide‐substituted PDI functional SMPs for application in electronic materials.

## Controlling Mechanism of Growth

**Figure 7:**
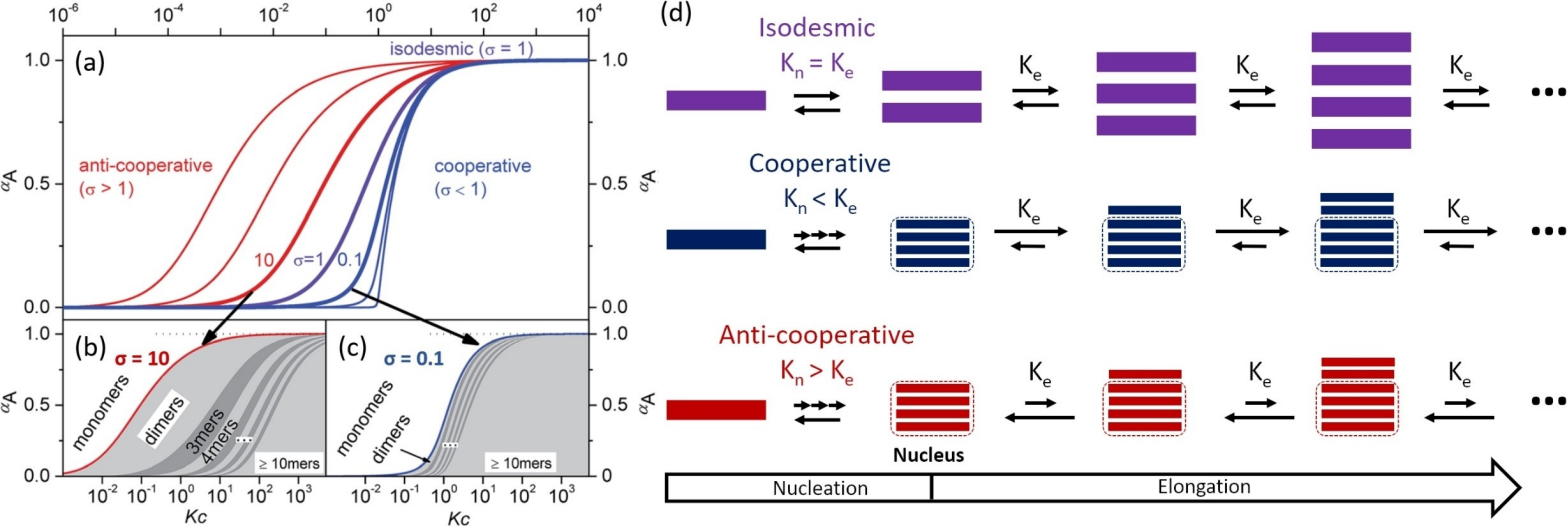
(a) Plot of aggregation fraction (*α_A_*) and concentration‐dependent *K_c_* with different *σ* (the cooperativity factor, *σ*=*K_n_*/*K_e_*). (b) Anti‐cooperative aggregation with *σ*=10. (c) Cooperative aggregation with *σ*=0.1. (d) Schematic representation of the *K_n_‐K_e_* model. Adapted from Ref. [58], licensed under a Creative Commons Attribution 3.0 Unported Licence. ‐Published by The Royal Society of Chemistry.

Supramolecular polymerisation can proceed through a variety of mechanisms, much like covalent polymerisation. Whilst the growth mechanism of covalent polymerisation is mostly dependent on the presence and chemistry of active sites, the growth mechanism of supramolecular polymerisation is dependent on the equilibria between successive growth steps ‐ for example, the equilibria between unimer and dimer, dimer and trimer, and *n*‐mer and *(n+1)*‐mer. Whilst each polymerisation will have its own set of equilibria, dependent on the unimer used and external factors such as solvent, three main growth mechanisms can be established: 1) isodesmic growth, where the equilibria between *n*‐mers are equal; 2) cooperative growth, where the equilibria between *n*‐mers becomes more favoured at a certain degree of polymerisation (DP_n_); and 3) anti‐cooperative growth, where the equilibria between *n*‐mers becomes less favourable above a certain DP_n_.^[57]^ These three supramolecular polymer growth mechanisms can be summarised through the *K_n_‐K_e_* model (Figure 7), which simplifies the equilibria between *n*‐mers in a growing polymer chain to two equilibrium constants ‐ nucleation (*K_n_*), and elongation (*K_e_*). Typically, the nucleation equilibrium is thought to occur over oligomeric length scales ‐ for example, *K_n_* is sometimes written as K_2_ (the equilibrium from unimers to dimers). Whilst this model was originally developed to describe cooperative growth, which has a defined nucleation stage (pre‐equilibrium),^[57]^ it can be used to describe all three growth mechanisms. Isodesmic growth exists where nucleation and elongation equilibria are indistinguishable (i. e., where *K_n_* =*K_e_*), resulting in a polymer whose binding strength (and thus elongation equilibrium) does not change with polymer length. As such, this, mechanism can be thought of as analogous to covalent step‐growth polymerisation, where growth is equally favourable, regardless of the DP_n_. Also, like step‐growth polymerisation, isodesmic polymers require extremely high concentrations to achieve high DP_n_ values.

Meanwhile, the elongation equilibria in cooperative growth systems are more favourable than nucleation equilibria (*K_n_* <*K_e_*), thus for the cooperative growth mode, the growth of existing nuclei into more stable polymers is thermodynamically preferred to the formation of new nuclei. This process is analogous to chain‐growth covalent polymerisation, and can be controlled in a similar manner as chain‐growth polymerisation by using ‘seeds’ (short polymers with a higher DP_n_ than nuclei) or chemically modified unimers as initiators for supramolecular polymerisation. This method, termed seeded growth, can be used to create supramolecular polymers with low dispersities. Cooperative growth favours the formation of extended polymers even at low unimer concentrations, analogous to chain‐growth polymerisation.

Alongside cooperative and isodesmic growth, a third mechanism of growth exists, not seen in covalent polymerisations: anti‐cooperative growth, where growth is less thermodynamically favoured as polymers increase in DP_n_. Anti‐cooperative growth favours the formation of smaller aggregates, even at high concentrations, and can even place a ‘ceiling’ on polymer length.[^57^, ^58^]

Another consideration is that, unlike covalent polymerisations, these supramolecular mechanisms of growth are not mutually exclusive, but instead exist in relative degrees, which can be quantified by the cooperativity factor, *σ*. The parameter *σ* describes the ratio of the nucleation constant to the elongation constant (*σ*=*K_n_*/*K_e_*) and by that the strength of cooperativity ‐ for example a system may be strongly cooperative with minimal nucleation (*σ*≪1), or weakly cooperative (*σ*<1) with many new nuclei forming during the process. Inversely, a process may be strongly anti‐cooperative and thermodynamically favour short polymers which do not grow above a certain DP_n_ (*σ*≫1), or it may be weakly anti‐cooperative and still yield extended aggregates despite the preference for nucleation (*σ*>1).^[58]^

### An illustration of extreme anti‐cooperativity in PDI aggregates

In simple, non‐hierarchical aggregates, clear design rules have been created and applied to favour cooperative or anti‐cooperative growth, promoting or suppressing elongation kinetics (*K_e_*, in the *K_n_‐K_e_* model).^[59]^ In the case of anti‐cooperative growth, utilisation of sterically demanding groups can disfavour large aggregates, as π‐stacking is hindered at the dimeric or oligomeric stage. An example of this behaviour was investigated by Shao et al., involving an asymmetric PDI bearing 3,5‐dodecyloxyphenyl and 2,5‐di‐*tert*‐butylphenyl groups (**PDI22**).^[60]^ Whilst the former motif alone did not hinder π‐stacking and was known to favour PDI aggregation when symmetrically substituted at the imide position, the latter displayed significant steric hindrance that disfavoured aggregation. Addition of a methyl spacer to the less demanding non‐substituted phenyl group allowed the PDI to take on a series of conformations with only one sterically accessible face of the perylene core to π‐stack with. As a result, the only aggregation step that could take place was dimerization, yielding a dimer that is too sterically shielded to undergo further stacking. Concentration‐dependent NMR and UV/Vis spectroscopy confirmed this behaviour, with an equilibrium favouring dimerization at higher concentrations. However, even at degrees of aggregation (*α*) above 90 %, no evidence for larger co‐facial aggregates was found.

### Tailoring PDIs for cooperative growth

Whilst anti‐cooperative growth is due to disfavoured aggregation of extended aggregates, cooperative growth can be driven by interactions that reinforce binding in larger aggregates, thus increasing *K_e_* relative to *K_n_*. One method to achieve this type of growth through the rational design of unimer species, is to utilise moieties that induce macrodipoles; the resultant macrodipole will then be stronger for oligomeric and polymeric aggregates as opposed to dimers. In a study of cholesterol‐bearing PDIs, Kulkarni et al. found that those synthesised with a dipolar carbamate linker (**PDI23** and **PDI24**) followed a cooperative self‐assembly mechanism, confirmed by temperature‐dependent UV/Vis spectroscopy investigations.^[61]^ However, when this carbamate linker was replaced by an ether (**PDI25**), the absence of the carbonyl dipole led to isodesmic growth. Furthermore, whilst all PDIs studied initially assembled into 1D polymers, the absence of a strong macrodipole for **PDI25** led to the formation of spherical aggregates as anisotropic growth could not be maintained over mesoscopic length scales. Thus, modification of the imide moiety (using a carbamate or ester linker) did not exclusively lead to changes in the mechanism of growth, but also influenced the morphology of PDI aggregates, revealing the limitations of this design strategy. Dielectric measurements confirmed the weak macrodipole found in **PDI25**, with *μ*=0.6 D, whilst carbamate‐linked **PDI23** and **PDI24** had dipoles an order of magnitude larger (*μ*=3–5 D). The importance of a macrodipole was reinforced when carbamate‐linked PDIs were examined with swallow‐tail alkyl substituents (**PDI26**) and were found to undergo isodesmic growth, with no macrodipole moment observed in dielectric measurements. Whilst the carbamate linker was present, the flexibility of the alkyl substituents in **PDI26** caused the aggregates to become too disordered to generate macrodipoles, and thus cooperative growth was suppressed. Therefore, to rationalise a unimer design for cooperative growth, the presence of macrodipole‐enabling moieties is required, as well as a structure, for example, rigid substituents and spacers, to ensure strong interactions between these moieties to create a macrodipole.

Cooperative growth can also be mediated via structural effects, whereby the formation of a structured polymer (e. g., a chiral helix) leads to additional stabilising interactions not observed in the oligomeric or seed state. Initially this phenomenon was applied to covalent polymerisations, such as the anionic polymerisation of triphenylmethyl methacrylate,^[62]^ and the β‐sheet to α‐helix conformational change of growing enantiopure alanine oligomers.^[63]^ In both cases, the formation of helical covalent polymers promoted different polymerisation kinetics, leading to a cooperative growth mechanism. Structural cooperativity has also been demonstrated in SMPs, such as oligo(phenylene vinylene)s, whose growth mechanism changes from isodesmic to cooperative as they assemble into helices at the *28‐mer* scale.^[64]^ Engelkamp et al., previously showed a chiral tuneable superhelix consisting of disk‐shaped molecules.^[65]^ The unimer was derived from phthalocyanine and modified by chiral alkoxyl disubstituted benzo crown ether moieties. Driven by π‐stacking, this molecule was found to form right‐handed helical substructures, coiling into left‐handed superhelices. The helical structures were however eliminated by introducing potassium ions, which dynamically inserted between the crown ether and restricted the conformation of phthalocyanines, causing chirality transfer to be blocked.

Although not formally explored to date, keeping these examples in mind, opportunities exist to exploit these design rules and explore the influence of helicity on the growth mechanism in PDI‐based SMPs.

### Growth mechanisms of multistage aggregates

Whilst the previous work in this section shows that the mechanism of growth can be and rationalised and tuned for non‐hierarchical aggregates, a more complex picture emerges for PDIs that undergo multiple stages of aggregation. For example, the asymmetric **PDI27** employed by Meijer et al. consisting of a 3,4,5‐dodecylphenyl group and a hydrogen atom substituent at the imide positions, appeared to show cooperative growth into H‐aggregates when examined with temperature‐dependent UV/Vis spectroscopy.^[66]^ This growth was characterised by the presence of a critical aggregation concentration. However, whilst cooperative self‐assembly should yield extended aggregates at higher concentrations, even at 2×10^−3^ M, a concentration two orders of magnitude higher than those used for the UV/Vis spectroscopy investigations, this system only formed aggregates of approximately 20–30 unimers, confirmed by SAXS measurements. On modelling the self‐assembly process, it was found that this behaviour could be explained with the anti‐cooperative model for aggregate elongation, but a cooperative initial step of self‐assembly for the presence of a critical concentration. It was postulated that the initial step, i. e., hydrogen bonding between the hydrogen‐substituted imide positions of the PDIs, led to the growth of the PDI dimers into 1D aggregates. The steric crowding of the dodecyl substituents at the corona of these aggregates disfavoured this elongation step, creating an anti‐cooperative system with an initial kinetic barrier to dimer formation. This theory was tested by *N*‐methylation of these asymmetric PDIs at their hydrogen‐substituted imide position. Upon methylation (to form **PDI28**), there was no critical aggregation concentration found, as the hydrogen bonding that enabled the cooperative step was no longer possible. Consequently, these aggregates were less stable than those made from unmethylated PDIs, highlighting the role of the hydrogen‐bonded dimers in stabilising the aggregate.

Whilst most examples discussed in this review involve covalently bonded moieties at the imide position, unsubstituted PDIs are excellent candidates for supramolecular ensembles owing to the rigidity of the imide group and the presence of two carbonyl and one N−H group (acting as hydrogen‐bonding donors and acceptors, respectively). Most notably, they are complementary with melamine derivatives, offering opportunity for a host‐guest bound complex at the imide positions. Thus, melamine derivatives can be used to selectively bind to the PDI imides, reversibly functionalising these positions and allowing for the growth‐controlling imide functionality (initially present on the melamine) to be switched ‘on’ or ‘off’ by binding or dissociating the melamine‐PDI complex. One of the first examples of these PDI‐melamine ensemble polymers, as studied by Würthner et al. two decades ago, consisted of a *bay*‐substituted (aryl, *tert*‐butyl or octyl) and hydrogen imide‐substituted PDI and an *N,N*‐alkyl disubstituted melamine (e. g., **PDI29**).^[67]^ With the alkyl‐substituted melamines providing amphiphilicity to the resulting PDI‐melamine complex, π‐stacked SMPs could be realised with a sterically demanding *tert*‐butyl *bay* substituent. Most strikingly, the polymerisation of this PDI follows a cooperative process, where hydrogen bonding between PDIs and disubstituted melamines of the form [AB]_n_ is required to form an ensemble, which could undergo π‐stacking‐mediated polymerisation. This mechanism of assembly was confirmed by disruption of the aggregates with monotopic *N*‐dialkyl substituted melamine, which could only bind to a single PDI unit and thus served as an end‐cap for the hydrogen‐bonded chain.

Expanding upon this research, Schenning et al. sought to develop this system further to introduce new electroactive moieties, namely oligo(*p*‐phenylenevinylene)s, to create hydrogen‐bonded triads by complexation with **PDI27**.^[68]^ The melamine‐functionalised oligo(*p*‐phenylenevinylene)s contained an extended π‐system to facilitate strong cooperative π‐stacking, allowing for defined ABA trimers to polymerise instead of the extended [AB]_n_ ensembles. As a result, this system polymerised upon binding of the oligo(*p*‐phenylenevinylene)s‐melamine moiety to the PDI terminus, which acted as the kinetic barrier (analogous to the nucleation step) to cooperative growth.

### Thermodynamic control of growth

In isodesmic systems, aggregate size is often dependent on concentration. Whilst in most PDI aggregates concentration alone is insufficient to control growth, extreme examples exist where aggregate lengths can be controlled unimer‐by‐unimer. One such example, examined by Echue et al., used the chiral (*S*)‐2‐N,N′‐dimethylamino‐3‐phenylpropanamine (DMAPAA) imide substituent, a bulky tertiary amine that can be further quarternised to an ammonium iodide salt (**PDI30**).^[69]^ These charged ammonium iodide salts then aided the perylene core in π‐stacking via ionic self‐assembly (ISA) with oppositely charged surfactants,^[70]^ creating one‐dimensional H‐aggregates. However, whilst temperature‐dependent UV/Vis spectroscopy confirmed the isodesmic growth model for these aggregates, the bulkiness of the ionised substituents hindered growth to dimer or trimer lengths at room temperature in H_2_O. The length of these aggregates could be finely controlled, with a linear relationship between concentration and calculated average stack length, ranging from 1×10^−5^ M (DP_n_=2.45) to 5×10^−5^ M (DP_n_=3.0).

One method for achieving thermodynamic control over polymer growth is the use of end‐capping molecules that bind and deactivate an active chain end, and thus suppresses further growth. If the end‐cap binds preferentially to the growing chain over new unimers, the introduction of these molecules can halt isodesmic, cooperative or anti‐cooperative growth, creating a thermodynamic minimum for the end‐capped, deactivated polymers. End caps are commonly used for hydrogen‐bonding aggregates, which can be easily modified from ditopic to monotopic unimers (i. e., end caps) through functionalisation of hydrogen‐bonding moieties. A prominent recent example of this has been Kang et al.’s *N*‐methylation of amides in corannulene‐based SMPs, yielding monotopic unimers, which serve as molecular initiators for cooperative supramolecular polymerisations.^[71]^ In addition, end‐capping has been trialled in a variety of supramolecular systems, including pyrimidone‐based unimers,^[72]^ urea‐functionalised calixarenes^[73]^ and ligating pyridine‐porphyrin complexes.^[74]^

**Figure 8:**
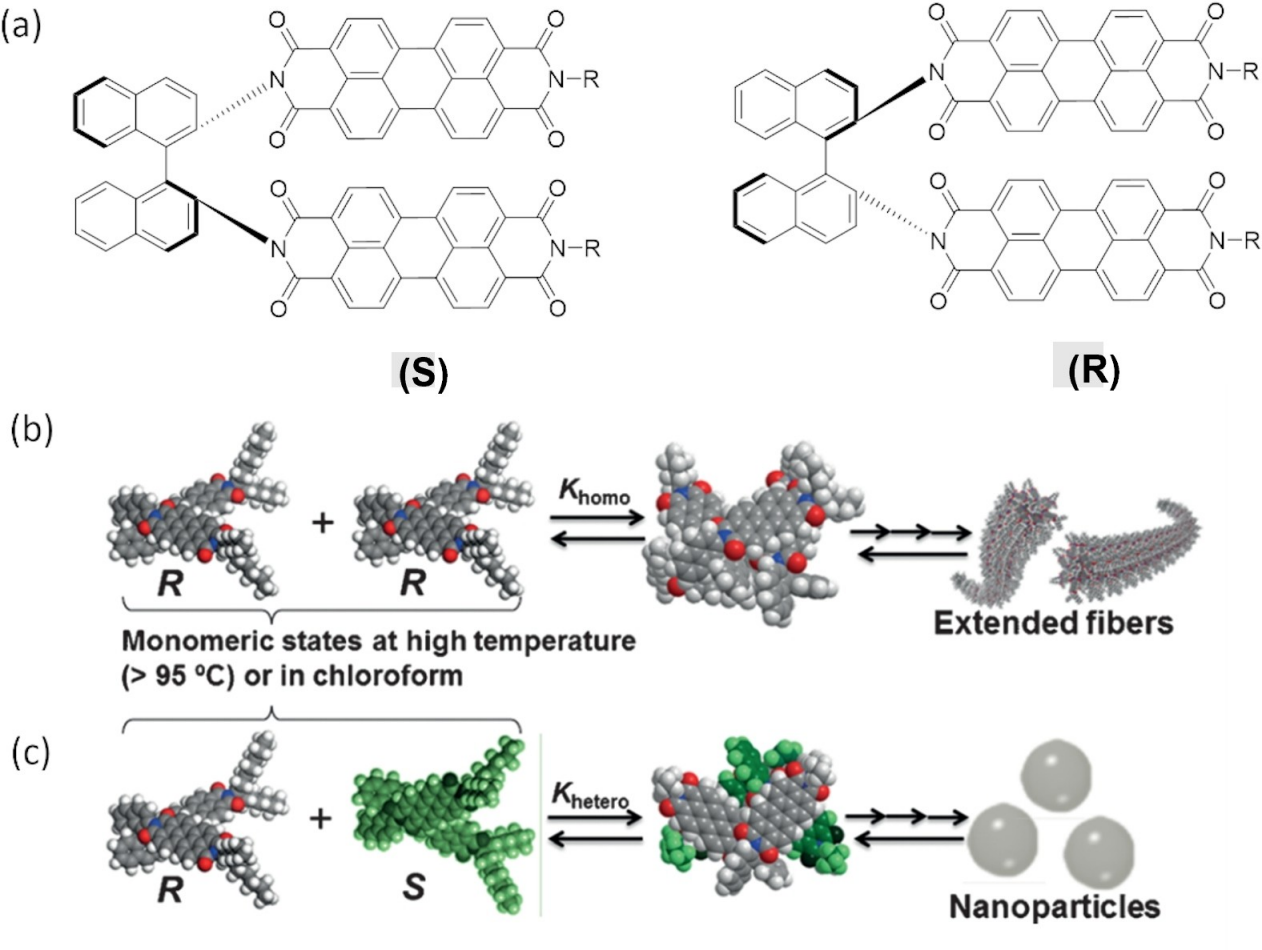
(a) Molecular structures of the S and R isomers of the chiral BINAP‐PDIs, and schematic illustration of their assembly in (b) homochiral and (c) racemic systems alongside their calculated dimeric models.^[76]^ Adapted from Ref. [76]. Copyright 2015, WILEY‐VCH.

There has been some promising work towards using end‐capping to control PDI SMP growth.^[75]^ For example, Kumar et al. explored the concept using a PDI dimer (BINAP‐PDI), bound via the imide positions to a chiral binaphthalene (BINAP) moiety.^[76]^ As such, these isomers exist in both *S* and *R* forms, which exhibit preferential heterochiral binding: whilst enantiopure samples formed defined nanowires, the racemic mixture of both BINAP‐PDIs formed thermodynamically favoured spherical aggregates (Figure 8). Both assemblies were found via temperature‐dependent UV/Vis investigations to undergo isodesmic self‐assembly processes, but the length of fibres could be controlled via changing the enantiomeric excess (*ee*) of either *R* or *S* isomers, with addition of the minor stereoisomer suppressing growth. However, this system does not exhibit true end‐capping, and appreciable length control could only be maintained through a narrow *ee* range. At low *ee* values (below 0.6), spherical nanoparticles predominated, whilst higher *ee* values (especially those above 0.8) led to more polydisperse mixtures as growing chain ends were not always successfully deactivated by the addition of a minor enantiomer, leading to populations of long fibres from failed deactivations coexisting with successfully controlled short fibres. Even with the limitations of this method, the ability to use enantiomeric excess alone to control growth, without affecting morphology (albeit within a narrow range of *ee* levels), is unprecedented. However, to achieve true end‐capping of PDI SMPs, the design of end‐capping molecules with only one growth site is a more promising route, as was shown very elegantly in the case of methylated corannulenes.^[71]^

Rather than using additives to slow the growth of aggregates, anti‐cooperative self‐assembly is an established mechanism of growth which can be harnessed to limit the growth of PDI aggregates. The anti‐cooperative mechanism alone offers little control over the size of PDI aggregates, however, by exploiting multistage self‐assembly, Gershberg et al. created a PDI derivative, **PDI31**, which preferentially self‐assembles into even‐numbered aggregates.^[58]^ To better understand the unusual solvent‐ and concentration‐dependent changes in the UV/Vis absorbance spectra of this PDI, the authors developed a new anti‐cooperative *K_2_‐K* aggregation model. This model predicts a remarkable preference for even‐numbered aggregates: even at high concentrations (10^−2^ M) in 3 : 7 methylcyclohexane/toluene mixture, 84 % of aggregates contained even numbers of PDI unimers, as determined by UV/Vis absorption data and respective mathematic model constructed for anti‐cooperative supramolecular polymerisation. The unimer, which utilises an α‐methylated amide linker at the imide position and 2,5‐dodecyloxyphenyl substituents to induce solvophilicity, strongly disfavoured unimer‐dimer additions, allowing dimer‐dimer additions to dominate in solution.

### Kinetic control of growth

Current research into the kinetically controlled growth of PDIs largely revolves around exploiting two phenomena: cooperative growth, and the use of metastable states. These two processes are of particular interest owing to their potential in facilitating living supramolecular polymerisation, a framework which requires a fixed number of active growth sites and a source of unimers that will selectively add to growing nuclei without forming new aggregates. In comparison to its more established counterpart, living (covalent) radical polymerisation, living supramolecular polymerisation provides fine control over SMPs with low dispersities by adjusting the ratio of supramolecular nuclei (seeds) and metastable unimers, termed the unimer : seed ratio. Practically, this approach offers an experimentally facile and reproducible method whereby fixed concentrations of unimers and seeds are combined and aged to yield polymeric aggregates with unprecedented length control.^[77]^ This method has been pioneered in other supramolecular systems, initially for high M_w_ polymer systems by Manners,^[78]^ and, in the case of low molecular weight systems, most notably by Ogi et al. using porphyrins^[79]^ and Kang et al. using corannulenes.^[71]^ In both the small‐molecule cases, cooperative growth is encoded for using amide moieties in peripheral chains. However, different mechanisms govern the fine control over aggregation kinetics; the corannulenes of Kang et al. facilitate living growth via a ‘deactivated’, intramolecular hydrogen‐bonded kinetically trapped state, whilst the porphyrins (studied by Ogi et al.) exhibit isodesmic off‐pathway J‐aggregation. Nevertheless, the function of these two mechanisms in realising living growth is the same ‐ they provide a reservoir of metastable molecules that add to cooperatively growing polymer chains without undergoing self‐nucleation.

Owing to the precedent of amide moieties being used to facilitate living polymerisations in other molecules, and their common use in PDI chemistry to facilitate cooperative growth, amide‐bearing PDIs present an ideal opportunity to explore living supramolecular polymerisation. One of the most extensively studied amide‐bearing PDIs, **PDI32**, initially pioneered by Li et al. as a versatile n‐type semiconducting organogelator, is able to aggregate in a range of solvents, including aromatic solvents, owing to intermolecular hydrogen bonding between amide groups.^[80]^ These gels are composed of networks of fibrous aggregates, with each helical fibre consisting of anisotropically π‐stacked, rotationally displaced unimers. Self‐assembly of these solutions at 10^−6^ M in chiral limonene resulted in control of the chirality of the resulting fibres ‐ aggregates in *R*‐limonene were left‐handed (*M*‐configured) whilst aggregates in *S*‐limonene were right‐handed (*P*‐configured).^[81]^ However, at higher concentrations (10^−4^ M) and faster cooling rates (to induce faster aggregation) this chiral bias was suppressed, implying that that self‐assembly process is governed by kinetic processes. Further work by Ogi et al. confirmed that these fibres followed a cooperative growth mechanism, aided by an additional kinetic trap.

**Figure 9:**
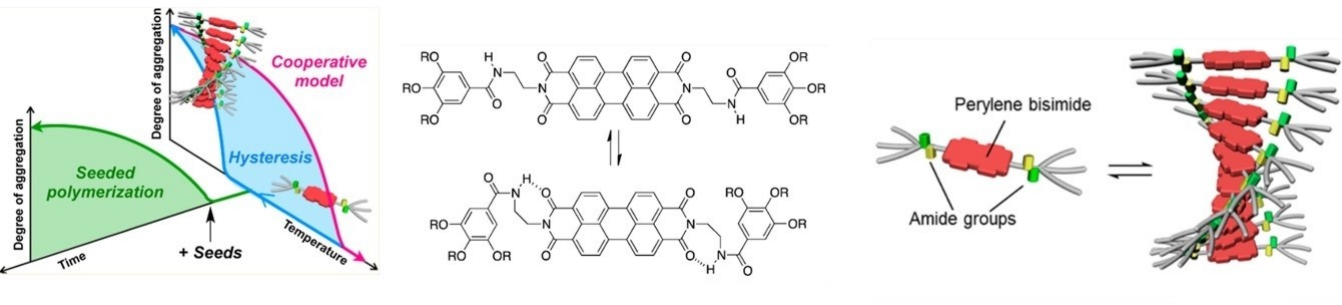
Overview showing living polymerisation, the equilibrium between open and closed conformations of **PDI22** and the self‐assembly of open **PDI22** into helical H‐aggregates. *R=n‐dodecyl*.^[83]^ Adapted with permission from Ref. [83]. Copyright 2015 American Chemical Society.

Spontaneous aggregation of unimers could be hindered by changing the length of the alkyl spacer between the PDI imide and the amide moiety, thus allowing for intramolecular hydrogen bonding between these two groups in a manner similar to the previously discussed corannulenes (Figure 9).^[83]^ This metastable conformation inhibited π‐stacking of the perylene chromophores; this conformation's stability could be readily modulated by concentration, temperature and solvent conditions, allowing for the metastable state to persist for up to an hour before polymerisation occurred. By observing the changes in UV/Vis absorbance, Würthner and co‐workers could follow the kinetics of aggregation. They demonstrated that the addition of polymer seeds to metastable unimer results in rapid aggregation, strongly suggesting the occurrence of living supramolecular polymerisation.

The capacity of this strategy to form uniform supramolecular structures was explored by the investigation of a structurally related PDI bearing 3,5‐oligo(ethylene glycol) aryl substituents by Manners and Faul.^[84]^ **PDI33** assembles cooperatively in polar solvents resulting in elongated nanofibers with a broad length distribution. Ultrasonication of these aggregates yielded sub‐100 nm nanofiber seeds with low length dispersities. The use of a binary common and selective solvent system resulted in a slow nucleation process, thereby allowing unimer to be added to these seeds with minimal formation of new fibres. By this method, highly uniform nanofiber suspensions with lengths ranging from 400–1700 nm and dispersities between 1.19 and 1.29 were formed. Significantly, a structurally similar PDI with alkene‐capped tethers, **PDI34**, was elongated from the same nanofiber seeds, resulting in supramolecular triblock copolymers. This copolymerisation process offers routes to more complex hierarchical PDI nanostructures with great potential for further emergent functionalities.

**Figure 10:**
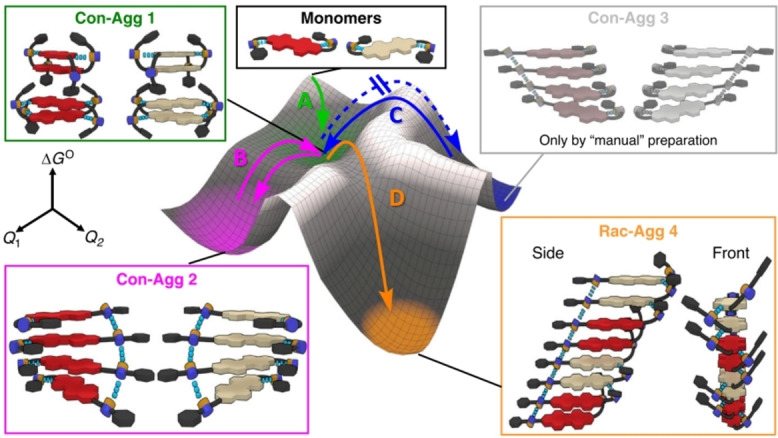
Energy landscape of the formation of conglomerate and racemic products starting with **PDI35/36**. Adapted with permission from Ref. [85]. Copyright 2020 Springer Nature. Creative Commons CC BY.

Another, more recent, example for kinetic control of the assembly‐behaviour was observed and explored by Wehner et al.^[82]^ They were able to observe the formation of three different aggregates of **PDI35** [(*S,S*)‐isomer] whilst maintaining the same solvent and unimer concentration.^[82]^ They were able to observe the formation of three different aggregates of **PDI35** whilst maintaining the same solvent and unimer concentration. Hereby, the first aggregate 1 (**Agg1**, pathway **A** in Figure 10) was formed and observed to be an on‐route state (direct formation of **Agg2** and **Agg3**, by pathways **B** and **C** respectively, without depolymerisation to unimers). The two following aggregates (**Agg2** and **Agg3**), of which **Agg2**, in the form of J‐stacked, one dimensional highly defined helical nanofibers, is the kinetically favoured and **Agg3**, also in the form of helical fibres but with a larger helical pitch, the thermodynamically more stable aggregate, could be achieved by ultrasonication at different temperatures and for different periods. The ability to obtain three different aggregates, led to those aggregates being labelled as “supramolecular polymorphs” by Würthner and co‐workers. In addition to analysis by CD, NMR, UV/vis and FTIR measurements, a variety of calculations were performed. By using thermodynamic and kinetic data obtained from temperature‐ and concentration‐dependent UV/vis measurements, an energetic landscape was simulated (Figure 10). In this energetic landscape the kinetic barrier for the dimer formation of **Agg2** and **Agg3** (pathways **B** and **C**, respectively) can be observed, as well as the energetically non‐hindered pathways of polymerisation initiated by the addition of **Agg2** and **Agg3** seeds. This example highlights once again how delicate balances can be exploited to gain control over SMPs.

Further research by Wehner et al. in 2020 showed even more control over the assembly of PDI‐based SMPs.^[85]^ They further explored different tools to influence the stacking behaviour of a racemic mixture of **PDI35** [(*S*,*S*)‐isomer and its (*R*,*R*)‐isomer], **PDI36**. Two conglomerates (homochiral aggregation products) and racemic (heterochiral aggregation product) were achieved by kinetic control and thermodynamic control, respectively. Treating a racemic mixture with the same conditions used to obtain **Agg1** and **Agg2**, homocoupling of both isomers could be shown by different analytic techniques. In contrast, no **Agg3** could be observed using the conditions applied before. However, after increasing the concentration by 25 % the formation of a new aggregate (**Agg4**, pathway **D**) was observed. In contrast to the helical structure seen in **Agg3**, no helices were observed for **Agg4**. The authors proposed an assembly of alternating (*S*,*S*) and (*R*,*R*) dimers, based on the amount of different observed interactions. This approach provided new insight into the assembly processes by providing an even more thermodynamically favoured aggregate with **Agg4**, as well as exploring the effect of symmetry breaking stacking and the cooperation of different isomers.

## Summary and Outlook

Achieving control of aggregation is a key challenge in the wider field of supramolecular chemistry, especially for SMPs. To realise the potential of PDI‐based SMPs in optoelectronic and sensing applications, careful design of PDI imide substituents is required. Such design has the potential to lead to fine control over the aggregation of these molecules and bestow additional functions for use, for example, in organic electronic devices. Keeping the fact in mind that the optoelectronic properties of PDI‐based materials are profoundly affected by their morphology, mode of stacking, and dimensions, the need for and importance of precisely controlling all aspects of growth and assembly becomes evident.

The collection of recent investigations examined here, clearly illustrate the design principles required to tailor the aggregation behaviour of imide‐substituted PDIs through the rational design and refinement of PDI unimers. These studies provide insight into ways to structure hierarchical PDI aggregates in the form of ensembles between PDIs and complementary binding molecules, or by using seeded growth to copolymerise PDI and as such PDIs provide an attractive platform towards developing these future technologies.

Within the field of PDI SMPs, much progress has been made towards controlling the structure and thus optoelectronic properties of these aggregates, including the realisation of dynamic and stimuli‐responsive systems. Design principles have been formulated to balance intermolecular interactions to achieve a variety of one‐dimensional SMP morphologies, including fibre, belt/ribbon and helical aggregates, and even incorporate them as key components of hierarchical structures such as superlattices as well as anisotropic 2D and 3D multicomponent aggregates. Whilst the formation of highly fluorescent J‐aggregated PDIs purely via imide substitution (i. e., without modifying the parent perylene chromophore) is challenging, nascent design principles for using steric bulk to favour J‐aggregation in PDI SMPs have been formulated. Moreover, the full potential of J‐aggregate control via imide substitution has recently come to light with the emergence of reversible, stimuli‐responsive J‐aggregation in PDIs, as well as Hj aggregates which exhibit spectral features of both aggregate types. The mechanism of growth of PDI aggregates can also be rationalised by various design principles, including the introduction of amide moieties to promote cooperative growth (also utilised in porphyrins and corannulenes) and the use of steric bulk to promote anti‐cooperative growth. Furthermore, thermodynamic and kinetic modes of control for these three growth mechanisms have been described, with a wide applicability to create equilibrium or out‐of‐equilibrium systems with controlled dimensions. Progress towards understanding the growth mechanisms of multistage (hierarchical or co‐aggregate) SMPs has also been made, with elucidation of these complex growth mechanisms used to predict the behaviour of specific systems.

Whilst several routes towards controlling the length and morphology of PDI SMPs exist and are covered by this review, in our opinion the most promising and widely studied method is that of seeded growth, which can produce polymers, block copolymers and other complex supramolecular architectures^[86]^ via living polymerisation. The requirement of a macrodipole to induce cooperativity, a key feature of seeded growth, can be selectively coded for via the addition of hydrogen‐bonding moieties such as amide linkers. However, to achieve living polymerisation, kinetic trapping is usually employed to further suppress nuclei formation, which often involves conformational changes or off‐pathway intermediate aggregates. Kinetic traps are difficult to design at the molecular scale, either requiring extensive computation to predict conformational changes or experimental analysis and screening of different PDI unimers. These kinetic traps are also highly dependent on polymerisation conditions (primarily solvent and temperature), limiting the processability options of living supramolecular polymers.^[83]^ However, such variations in conditions do provide opportunities to engage with automation, and using existing data sets, to explore optimised conditions to produce targeted structures and function.

Nevertheless, the potential for any imide‐substituted PDI to undergo living growth with the correct dipolar motifs and polymerisation conditions highlights the fact that these molecules are ideal candidates for nanostructured materials and devices, and offer a tantalising future where complex SMP‐based materials can be fabricated through chemical functionalisation, classical covalent chemistry and fully controlled self‐assembly.

## Conflict of interest

The authors declare no conflict of interest.

1

## Biographical Information

*Robert Wilson‐Kovacs received his MSci in Chemistry at the University of Bristol (UK) in 2018. Inspired by his final‐year MSci research project with the Faul Research group, he went on to undertake an MRes in Chemistry with the group, obtaining it in 2020. Currently, he is undertaking a PhD with the Olivier Research group at the University of Miami (Florida, USA). His research focus is the controlled self‐assembly, post‐assembly chemical modification and optoelectronic characterisation of functional supramolecular polymers*.

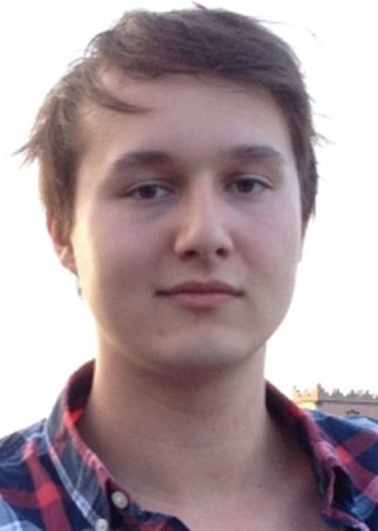

## Biographical Information

*Xue Fang completed her BSc in Chemistry at ShanghaiTech University (Shanghai, China) in 2019. Supervised by Prof. Charl F.J. Faul, she then obtained the degree of MSc by Research in chemistry in 2020 at the University of Bristol (UK). Since then, she continued with PhD in chemistry in the Faul Research group. Her research focus is design and control of functional supramolecular polymers*.

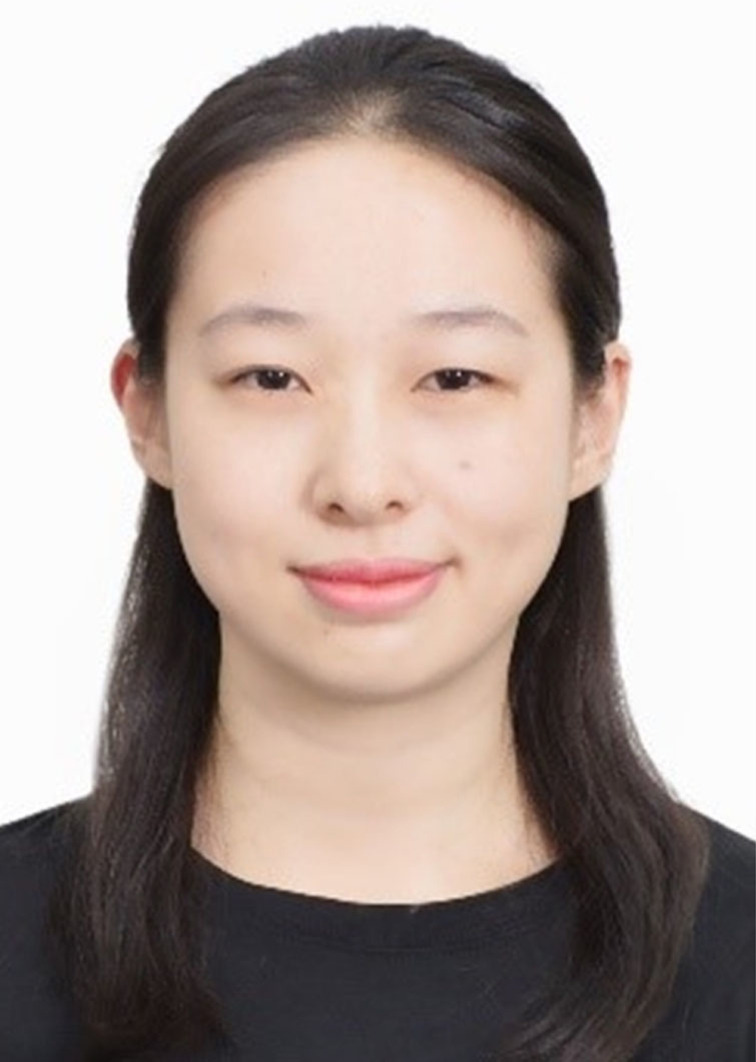

## Biographical Information

*Maximilian Hagemann obtained both his BSc in Chemistry in 2017 and his MSc in Chemistry in 2019 at the University of Münster, Germany. During his master he did an Erasmus internship on the synthesis and characterisation of corannulene‐based supramolecular polymers in the group of Prof. Faul at the University of Bristol, England, UK. He re‐joined the Faul Research group in November 2019 as a PhD student. His work focuses on controlling functionality and self‐assembly of PDI‐based supramolecular polymers by targeted chemical modification*.

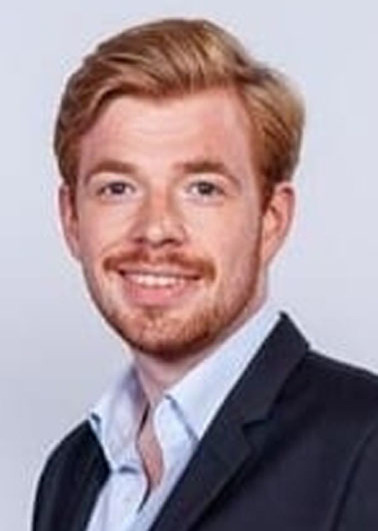

## Biographical Information

*Henry E. Symons graduated from the University of Bristol in 2015 with an MSc degree in chemistry. He completed a PhD under the supervision of Prof. Charl F. J. Faul on the kinetically‐controlled formation of functional supramolecular polymers. During his studies, he investigated topics including living supramolecular polymerisation, heteroatom substitution, and covalent post‐modification. Since graduating in 2021, he has carried out post‐doctoral research relating to the COVID‐19 pandemic and on protocellular materials*.

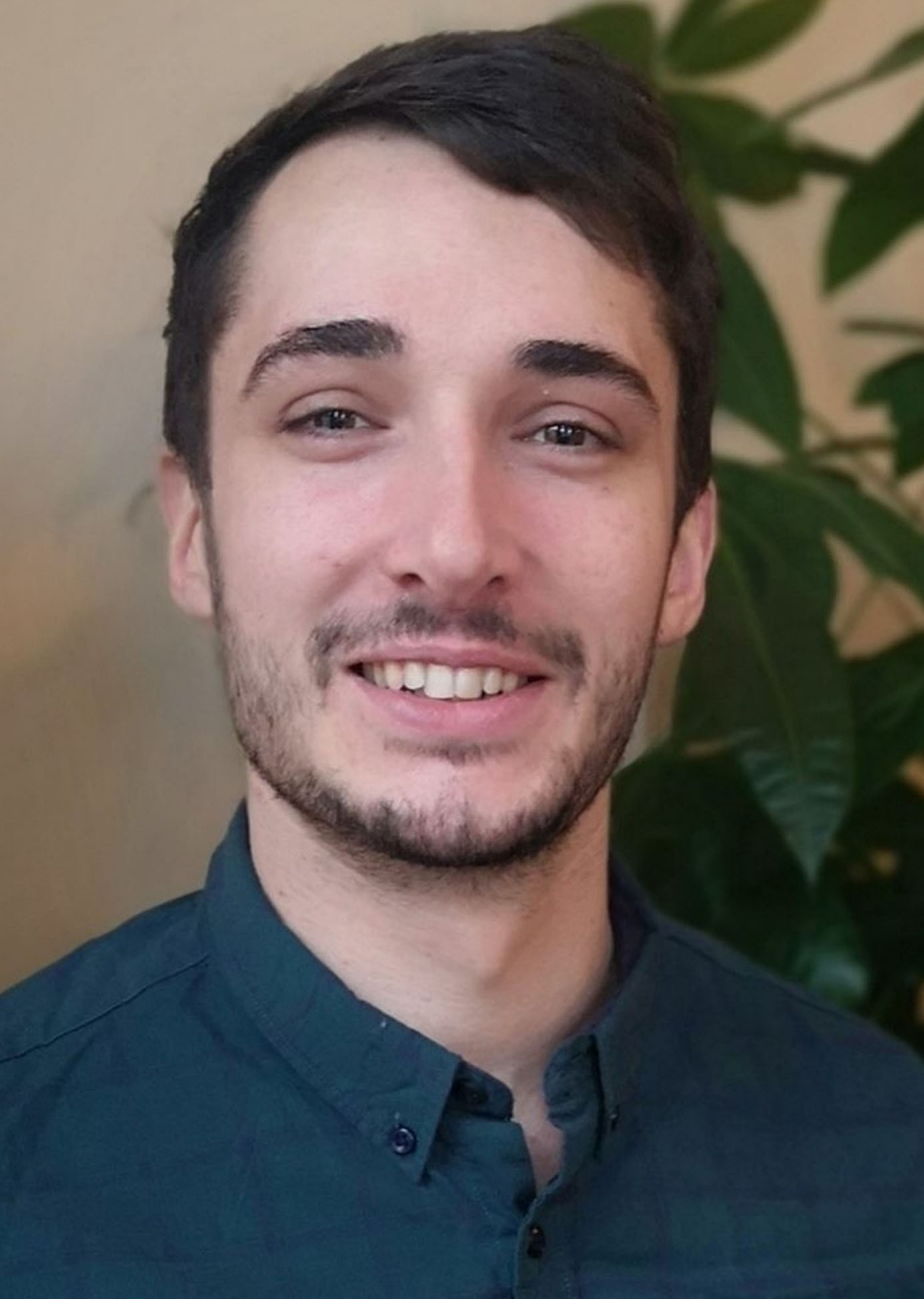

## Biographical Information

*Charl F. J. Faul received his PhD in 2000 from the University of Stellenbosch, South Africa. After working for a year as postdoctoral research fellow with Markus Antonietti at the Max‐Planck‐Institute of Colloids and Interfaces (Potsdam, Germany), he led a group exploring ionic self‐assembly until 2004 at the same institute. He then moved to the School of Chemistry, University of Bristol, where he is currently a Professor of Materials Chemistry. His research explores the design principles and applications of electroactive porous and supramolecular systems*.

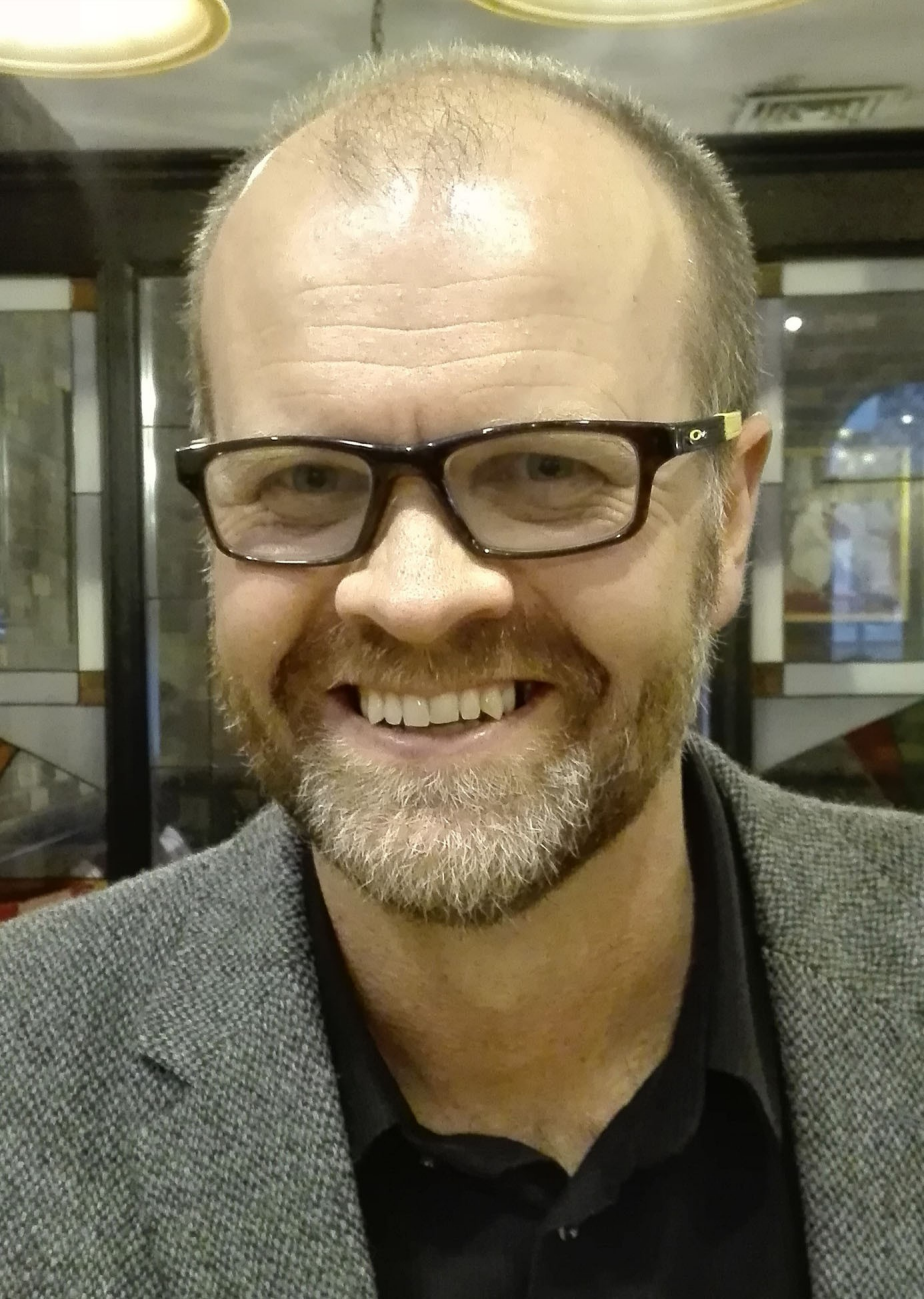

## Data Availability

This study did not involve any underlying data.
